# Modeling the traffic flow of Vakil Abad Boulevard in Mashhad at the intersection of Kosar and ‎presenting proposed solutions

**Published:** 2019-08

**Authors:** Mohammad Ebrahim Amin Ghafoori, Bahram Farhadinia, Mahbobeh Banaee Kariz Bala

**Affiliations:** ^*a*^Mashhad Municipality, Quchan University of Technology, Mashahd, Iran.; ^*b*^Quchan University of Technology, Iran.; ^*c*^Hakim Sabzevari University, Sabzevar, Iran.

## Abstract

**Background:**

The main axis of communication between the east and west of Mashhad is Mellat Park Square. ‎Increasing travel demand from the Vakil Abad axis creates heavy traffic at the intersection of ‎Kosar. The existence of the metro station at this intersection and its interference with taxi and ‎bus stations will exacerbate traffic and insecurity for the citizens. By modeling this point, based ‎on the Google Earth Maps, we outline the traffic situation and the factors that affect it. To ‎improve the situation, we will model the scenario of changing the location of metro, taxi and ‎bus stations, and analyze the results. Targets: Understand the status quo modeling the current ‎situation Identify incident points Provide a solution along with a model designed to improve ‎conditions. ‎

**Methods:**

Data collection and analysis with software SPS and esy fit Use Google Earth Maps for modeling ‎Simulation with software Anylogic. ‎

**Results:**

Inappropriate location of the metro station at the intersection of Kosar Incorrect Bus Station at ‎the intersection of Kosar Inappropriate location of bus stations after the intersection of Kosar. ‎

**Conclusions:**

The transfer of the subway station to 50 meters along Kowsar Avenue, the transfer of the taxi ‎station to the intersection and with the development of the trough at the intersection of Kosar to ‎the University of Trosi Bus stations will be better placed and will increase the safety of citizens ‎and reduce traffic.

**Keywords:**

Traffic, Safety, Vakil Abad, Station

